# Prospective Randomized Controlled Study of Hemostatic Efficacy with Kaolin-Impregnated Dressings in Diabetic Foot Ulcers Taking Anticoagulants Undergoing Debridement in an Outpatient Clinic

**DOI:** 10.1155/2019/9316380

**Published:** 2019-11-11

**Authors:** Yeok Gu Hwang, Jin Woo Lee, Eun Ae Won, Seung Hwan Han

**Affiliations:** ^1^Department of Orthopedic Surgery, Ewha Womans University Seoul Hospital, Ewha University College of Medicine, 260, Gonghang-daero, Gangseo-gu, Seoul 07804, Republic of Korea; ^2^Department of Orthopedic Surgery, Severance Hospital, Yonsei University College of Medicine, 50-1, Yonsei-ro, Seodaemun-gu, Seoul 03722, Republic of Korea; ^3^Department of Nursing, Gangnam Severance Hospital, Yonsei University College of Medicine, 211, Eonj-ro, Gangnam-gu, Seoul 06273, Republic of Korea; ^4^Department of Orthopedic Surgery, Gangnam Severance Hospital, Yonsei University College of Medicine, 211, Eonju-ro, Gangnam-gu, Seoul 06273, Republic of Korea

## Abstract

**Background:**

The effective hemostasis after minor debridement in an outpatient clinic is important and essential. This study is aimed at evaluating the hemostatic efficacy and safety of the kaolin-impregnated dressing for diabetic foot ulcer patient who take an anticoagulant.

**Methods:**

A prospective, randomized, clinical trial study was performed in twenty-six patients with diabetic foot ulcers who take an anticoagulant requiring minor debridement in an outpatient clinic. Minor debridement and removal of break down skin were performed by one orthopedic surgeon. Hemostasis on wound bed was achieved using kaolin-impregnated gauze (study group) and standard sterilized dry gauze (control group). Two randomized groups were compared for hemostatic efficacy and side effect.

**Results:**

For the purpose of this study, the presence or absence of hemostasis was assessed at 5 and 10 minutes after the application of dressing material. Treatment was evaluated as successful if bleeding was ceased adequately, and no extra hemostatic measures were required within 10 minutes. At 5 minutes, 80% of patients using the kaolin-impregnated gauze successfully achieved complete hemostasis versus 40% in the control group that controlled bleeding partially. With kaolin-impregnated gauze, 100% of patients show complete hemostasis at 10 minutes versus 58.3% in the standard gauze (*P* < .001). An adverse effect was not noted in all patients.

**Conclusions:**

The use of kaolin-impregnated gauze appears to be a safe and feasible option in managing bleeding after debridement of diabetic foot ulcers, and merits to patient who had a bleeding tendency is high. This trial is registered with CRIS registration number KCT0003894.

## 1. Introduction

Repeated minor debridement for superficial or deep diabetic foot ulcer with infection is an essential procedure to remove necrotic tissue including the bone, abscess, and provides a low rate of local complications [1]. Removal of the infected bone or break downed skin during minor debridement based on an outpatient clinic is conventionally followed on external compression achieved with a simple sterilized gauze applied to the debrided ulcer site [2]. Besides, the vasculopathy is a common complication of diabetic patient, who have to receive anticoagulation therapy for protecting cardiovascular attack and atherosclerotic change of vessels. In diabetes patient, a propensity for platelet activation and aggregation, coupled with a tendency for coagulation, is relevant to a risk of thrombosis complicating plaque rupture [3]. With those conditions, hemostasis after debridement is relatively important but usually considered as a suspended complication of aggressive procedures because of the small amount of bleeding. However, the bleeding tendency of diabetic ulcer increases due to anticoagulation therapy. Hence, there is a growing interest to reduce hospital stay after interventional procedures and to find alternative bleeding control methods to avoid collagen-based or suture-based hemostasis in patient settings, without increasing procedural complications.

Currently, there are many studies that reported effectiveness after kaolin-impregnated dressing in trauma [4, 5] and percutaneous vascular intervention [6, 7], but the advantages in terms of efficacy of these materials compared to conventional sterilized gauze have never been demonstrated for hemostasis in diabetic foot ulcer undergoing surgical debridement based on an outpatient clinic.

The purposes of this study were to examine the effectiveness and safety to evaluate the hemostatic effect of kaolin-impregnated gauze on diabetic foot ulcer patients who continuously take anticoagulants, comparing with a control group on hemorrhage control after debridement in a diabetic foot ulcer [8].

## 2. Patients and Methods

All consecutive patients undergoing major or minor debridement or superficial necrotic tissue removal procedures between March 31, 2016 and June 22, 2016 at an outpatient clinic of a university hospital were considered to be enrolled in the study. After that, the patients were randomized in a 1 : 1 fashion to conventional manual compression methods with sterile dry gauze or kaolin-impregnated gauze for achieving hemostasis. Randomization was based on a computer-generated randomization list. All patients were enrolled to the study with the following inclusion criteria: (1) patients over 20 years old who have type I or II diabetes mellitus (DM), (2) an ulcer was Wagner grade I or II ulcer, (3) above 2 cm diameter size which lasted more than six weeks without infection or inflammation (local tenderness, erythema, generalized fever, and leukocytosis), (4) good extremity circulation status, palpable pulse, or confirmation with Doppler sonography at the ankle (dorsalis pedis artery or posterior tibial artery), (5) received anticoagulation therapy due to diabetic vasculopathy or systemic disease, and (6) patients who agreed the written consent form after full description of the clinical trial. Patients that were excluded from the study were pregnant patients, patients undergoing immunosuppressant therapy, and patients with systemic cardiopulmonary disease. Also, patients who does not understand or agree to this study were excluded.

### 2.1. Sample Size Calculation

A previous similar study showed that received chitosan dressings reduced survival time compared with gauze sponges [9]. The average success rate of hemostasis after 5 minutes was acquired 0.1% (gauze sponges) and 62% (chitosan-based hemostatic dressing), respectively. Similarly, the sample size estimate for the present study was 13 patients in each group based on the following calculation. Assuming *α* = 0.05 and 1‐*β* = 0.9 and that the ratio of the two groups is 0.001 and 0.620, it was calculated as 10 patients for each group. We chose to include 26 patients (13 in each group) to adjust for an estimated 20% dropout rate [10] (PASS version 15, NCSS, LLC, Kaysville, Utah, USA).

### 2.2. Study Design

A total of 26 patients enrolled to find out a statistically significant difference in the complete bleeding control between the two groups. At first visit, qualified patient was distinguished based on physical examination along with foot radiography and routine laboratory test (complete blood count, routine chemistry screen, urinary analysis, and vital sign checkup), and previous medical records were reviewed. Those patients were randomized into the two different groups with a 1 : 1 ratio. After achieving the fresh bleeding on the wound bed and based on a computer-generated randomization list, the patients were allocated into one of the following strategy groups. Study group: the compression of wound bed was obtained with kaolin-impregnated gauze applied directly after procedure. Control group: hemostasis was obtained by direct compression of wound bed with a folded conventional sterile gauze wrapped with tape and maintained for 5 minutes while patients in the control group were managed with conventional manual compression dressing using dry gauze (sterilized gauze, DaeHan Medical, Chungju, South Korea) after procedures. During the treatment, the anticoagulation therapy is continued as usual. Moreover, to observe, a side effect, either reported by the patients or inspected by the investigator, was checked using a standardized protocol through the study.

### 2.3. Product Description

Kaolin-impregnated hemostatic dressing is flexible, nonwoven coated gauze (50-50 rayon/polyester, 2^∗^2 inch). Kaolin is an aluminum silicate, a very potent coagulation initiator that acts as a surface activator with a hemostatic effect caused by the activation of the intrinsic clotting pathway [11, 12]. The gauze is stable after opening the external aluminum envelope. It is absorbent and has a good clotting ability. This advanced clotting gauze is a Food and Drug Administration (FDA) and Korean Food and Drug Administration (KFDA) cleared device.

### 2.4. Study Protocol

**Figure 1:**
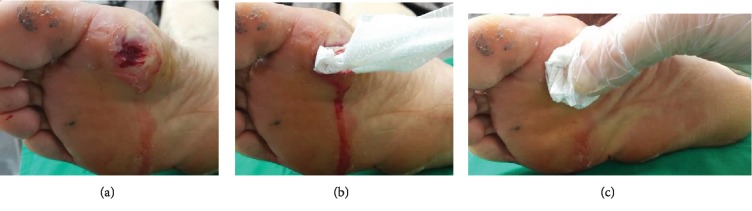
Serial hemostatic procedures of conventional manual compression dressing using a kaolin-impregnated dressing (study group) or dry gauze (control group) for a diabetic foot ulcer after surgical debridement. (a) Devitalized margin of the diabetic foot ulcer was debrided using a surgical blade. (b) Application of kaolin-impregnated dressing after soaking to the ulcerative wound using sterilized forceps. (c) Manual compression with constant pressure.

The basic dressing protocol was standardized as follows (Figure 1):

1. Through cleansing of the wound bed and margin with normal saline
2. Additional debridement to remove necrotic tissues and expose healthy bleeding margin if necessary
3. Direct application of the dressing material to wound bed then compress with constant pressure manually for 5 minutes by a trained registered nurse who was specialized in wound care
4. At the time of estimated 5 minutes, the presence or absence of hemostasis was assessed at that time
5. Test treatment was repeated if hemostasis was not achieved after 5 minutes. Hemostasis was assessed again at 10 minutes after treatment starts

### 2.5. Hemostatic Endpoint

The definition of hemostatic endpoint was the complete stopping status of bleeding on the debrided wound bed, and no further compression was required to control bleeding at the debridement site [6]. The treatment was rated as effective if bleeding was stopped sufficiently, and no additional hemostatic measures were necessary within 10 minutes. The bleeding tendency and complication rate was evaluated and analyzed. Patients were evaluated at 5 minutes and 10 minutes after hemostasis achievement for complications.

### 2.6. Statistical Analysis

Patient characteristics and clinical outcomes are presented as mean ± SD or count (percentage). We first performed the Shapiro-Wilk normality test to check for normal distributions of study variables. If normality tests were passed, an independent *t* test was used. If normality tests were failed, the Mann-Whitney test was used for comparisons after treatment. Statistical analyses were performed using SPSS software (Version 18.0, IL, Chicago, USA). A significance level of 5% was used for all analyses.

### 2.7. Ethical Considerations

Approval to conduct the study was given by the Gangnam Severance Hospital, Institutional Review Board (No. 3-2014-0083) in accordance with the Declaration of Helsinki, and written informed consent was obtained from all patients prior to the study.

## 3. Results

### 3.1. Demographics

Of the enrolled 26 patients (13 for each group) in the study and followed for endpoints, two patients were stopped because of a high rate of active bleeding after debridement of all necrotic tissue and surrounding callus. Subsequently, the randomization was done with 12 patients. The demographics and medical history of the two groups are presented in Table 1. Initial diabetic foot ulcer status also compares with two assigned groups regarding the period of morbidity of diabetic foot ulcer, ulcer size, and grade in Table 2. No major adverse events such as uncontrolled major bleeding, acute infection sign, and newly developed ulcers were observed.

### 3.2. Safety and Efficacy

Safety of the studied dressing materials was evaluated by the percentage of patients experiencing complications defined as rebleeding or hematoma. An adverse event was defined as acute local infection, either clinical or in laboratory results with a minimum follow-up of 8 weeks.

### 3.3. Clinical Result of Hemostatic Endpoint

The use of kaolin-impregnated gauze resulted in a significant improvement of the time to complete hemostasis and overall hemostatic success. Patients treated with the kaolin gauze successfully achieved complete hemostasis at 5 minutes in 80% of cases, whereas only 40% of patients in the control group had partially stopped bleeding (*P* = .001). At 10 minutes, 100% of patients of the study group had hemostatic success versus 58.3% in the control group (*P* < .001). Thus, hemostasis was achieved after a study protocol in all of the study group and three patients in the control group. In two remaining patients who did not acquire hemostasis, we applied more compression time to the wound with bandage. Analysis of bleeding tendency through coagulation profiles of the two groups is presented in Table 3. Neither rebleeding nor hematoma occurred after the removal of dressing materials. Patients were on aspirin (6/24, 26.3%), clopidogrel (5/24, 20.8%), aspirin and clopidogrel (11/24, 45.8%), aspirin and LMHW (1/24, 4.2%), and warfarin (1/24, 4.2%). The type of anticoagulants of each group of patients is presented in Table 4.

### 3.4. Clinical Follow-Up

A telephone contact at 1 month was done to two patients (one each group) who have experienced a high rate of active bleeding after debridement. No major events on wound occurred.

## 4. Discussion

This study demonstrated that kaolin-impregnated gauze is a useful option to apply quick hemostasis for debrided diabetic foot ulcer. For standardized treatment to diabetic foot ulcers, currently many other therapeutic interventions existed: off-loading, dressings, topical therapies, electrophysical therapy, negative pressure wound therapy, platelet-rich plasma and surgical debridement, and so on. Among them, debridement is essentially considered a part of diabetic foot ulcer standard care [13]. It arise removal of callus and abnormal margin tissue and necrotic portion and reduction of bacterial biofilms and excess matrix metalloproteinases (MMPs) [14]. However, as we described above, the bleeding tendency of ulcer increases due to anticoagulation therapy in diabetes patients.

For overcoming those problems, we suggested that new developed clotting materials to control massive bleeding at combat field would be applicable to diabetic foot ulcer with the same goal. The kaolin-impregnated gauze is known as combat gauze that shows effective hemostasis of the external bleeding in combat field. To avoid difficulty in achieving hemostasis in trauma and interventional procedures, advanced materials impregnated with kaolin have been introduced with good results [15, 16]. It used the first-line hemostatic agent for use in treatment of severe hemorrhage that cannot be controlled by a tourniquet [17].

Kaolin-impregnated dressing is an inert zeolite-based mineral substance. It is a subsidiary of volcanic rock reproduced in the laboratory and acts as a selective sponge been tested in animal models [18]. When it comes in to contact with blood, the zeolite rapidly takes the smaller water molecules from the blood into its numerous pores leaving a cellular clot behind [19]. This process of physical adsorption is accompanied by an exothermic reaction which generates temperatures of up to 105°F [20]. These findings increased expectation to those hemostatic materials which might promote clotting by activation of factor XII (FXII) and factor XI (FXI) of the intrinsic coagulation pathway by providing more optimal surrounding. Previous in vivo animal hemorrhage models found the kaolin-based gauze to be the most effective product among four new dressings tested [16]. Traditionally conventional manual compression dressings using folded dry gauze are used for the control of bleeding from diabetic foot ulcer after surgical debridement. However, it is difficult to manage a continuous oozy bleeding from that lesion; furthermore, anticoagulant thins the blood. Limited data exist relative to the effectiveness of hemostatic agents when the patient suffers from diabetic foot ulcer. Currently, under the author's knowledge, no study reported hemostatic effectiveness and safety with diabetic foot ulcer after debridement in an outpatient clinic. Besides, the conventional manual compression dressing to control a bleeding is well-known, but the use of hemostatic dressing containing kaolin can be a new and simple approach to those, either ongoing or after surgical debridement. In this study, the authors recommend a user-friendly and easy outpatient clinic treatment protocol for diabetic foot ulcer management with postdebridement bleeding; hemostasis rate is 80% in a 5-minute treatment and 100% within a 10-minute treatment.

The current study has a few limitations. Nevertheless, the two groups displayed similar demographics (Table 1) and coagulation ability (Tables 3 and 4); a small number was recruited due to limited study and budget. However, in this circumstance, as a strength in the design, a proper sample size was done before we investigated this study. Second, the investigator was not blind to the study materials because of different figure. To avoid the bias, hemostatic endpoint was checked by an independent observer who was not related to this study. Additionally, only the presence or absence of hemostasis was assessed at planned 5 and 10 minutes after treatment starts. Lastly, a different anatomical site of diabetic foot ulcer is involved to the current study. It might affect the amount and velocity of bleeding. Despite some limitations, we believe the prospective, randomized study design might add objective to the result of this study.

## 5. Conclusions

The present randomized study demonstrated that a hemostatic effect based on kaolin-impregnated dressing material combined with gentle manual compression for a few minutes is significantly superior to a conventional technique with dry gauze in acquiring the hemostasis after debridement procedures.

## Figures and Tables

**Table 1:** Demographics and initial evaluations of patients.

	Control (*n* = 12, 50.0%)	Study (*n* = 12, 50.0%)	*P* value
Age (years)	58.9 ± 11.5	68.7 ± 10.8	.065
Gender (men : women)	7 : 5	6 : 6	
Body mass index (kg/m^2^)	24.6 ± 2.7	24.1 ± 4.3	.773
Systolic BP (mmHg)	136.0 ± 18.3	134.0 ± 21.2	.823
Diastolic BP (mmHg)	70.9 ± 11.3	73.0 ± 8.2	.640
Heart rate (/min)	82.7 ± 12.1	77.1 ± 16.6	.400
White blood cells (×10^3^/*μ*l)	7.5 ± 3.7	6.5 ± 1.6	.089
HbA1c (%)	8.8 ± 2.9	7.9 ± 1.4	.426
Duration of diabetes (years)	15.9 ± 7.5	22.8 ± 12.4	.157
Smoking			
Nonsmoker	23 (74.2%)	16 (51.6%)	
Former smoker	5 (16.1%)	14 (45.2%)	
Smoker	3 (9.7%)	1 (3.2%)	

**Table 2:** Comparison of ulcer characteristics between the two groups.

	Control (*n* = 12, 50.0%)	Study (*n* = 12, 50.0%)	*P* value
Wagner ulcer classification			.248
Grade 1	2 (16.7%)	1 (8.3%)	
Grade 2	10 (83.3%)	11 (91.7%)	
Site			.791
Forefoot	6 (50.0%)	8 (66.7%)	
Midfoot	3 (25.0%)	3 (25.0%)	
Hindfoot	3 (25.0%)	1 (8.3%)	
Ulcer area (cm^2^)	19.33 ± 17.39	14.30 ± 9.25	.431

**Table 3:** Characteristics of common coagulation studies.

	Control (*n* = 12, 50.0%)	Study (*n* = 12, 50.0%)	*P* value
PT (sec: 9.2~12.3^∗^)	12.36 ± 1.37	13.16 ± 1.83	.283
PT (INR: 0.91~1.16^∗^)	1.05 ± 0.11	1.12 ± 0.14	.227
aPTT (sec: 26.8~40.6^∗^)	36.22 ± 22.16	37.15 ± 13.42	.911

^∗^Reference range.

**Table 4:** Type of anticoagulants of each group of patients.

	Control (*n* = 12, 50.0%)	Study (*n* = 12, 50.0%)
Aspirin only	3 (25.0)	3 (25.0)
Clopidogrel only	2 (16.7)	3 (25.0)
Aspirin and clopidogrel	6 (50.0)	5 (41.7)
Aspirin and LMWH	1 (8.3)	0 (0)
Warfarin	0 (0)	1 (8.3)

LMWH: low molecular weight heparin.

## Data Availability

The data used to support the findings of this study are available from the corresponding author upon request.
